# The Destruction of Rats by Means of an Infectious Disease

**Published:** 1900-09

**Authors:** 


### The Destruction of Rats by Means of an Infectious Disease.

J. Danysz describes in *Annales de l'Institut Pasteur,* April, 1900, his experiments with a bacillus fatal to rats, but harmless to man and the domestic animals. This organism is grown upon agar, and being spread upon bread, and placed in situations frequented by rats, is eaten by them in preference to most other foods. The resulting sickness appears in a few days, is usually fatal, and is communicated to other rats, either directly by the dead bodies which are devoured, or through the medium of food and drink polluted by the sick rats. It is, of course, necessary to maintain a virulent organism, in order to apply this agent to the destruction of rats. Danysz finds that the organism can be raised to a high virulence by intraperitoneal cultivation in guinea-pigs. The rats which were subjected to this infection in Danysz's experiments were Rattus and Decumanus.—*Maryland Medical Journal.*

